# Hyperglycemia Altered DNA Methylation Status and Impaired Pancreatic Differentiation from Embryonic Stem Cells

**DOI:** 10.3390/ijms221910729

**Published:** 2021-10-03

**Authors:** Andy Chun Hang Chen, Wen Huang, Sze Wan Fong, Chris Chan, Kai Chuen Lee, William Shu Biu Yeung, Yin Lau Lee

**Affiliations:** 1 Shenzhen Key Laboratory of Fertility Regulation, Reproductive Medicine Center, The University of Hong Kong, Shenzhen Hospital, Shenzhen 518000, China; andycch0@hku.hk; 2 Department of Obstetrics and Gynaecology, Li Ka Shing Faculty of Medicine, The University of Hong Kong, 21 Sassoon Road, Hong Kong; huangw14@connect.hku.hk (W.H.); szewan11@hku.hk (S.W.F.); chanwhchris@gmail.com (C.C.); bolkcad@gmail.com (K.C.L.)

**Keywords:** hyperglycemia, human embryonic stem cell, DNA methylation, pancreatic differentiation

## Abstract

The prevalence of type 2 diabetes (T2D) is rapidly increasing across the globe. Fetal exposure to maternal diabetes was correlated with higher prevalence of impaired glucose tolerance and T2D later in life. Previous studies showed aberrant DNA methylation patterns in pancreas of T2D patients. However, the underlying mechanisms remained largely unknown. We utilized human embryonic stem cells (hESC) as the in vitro model for studying the effects of hyperglycemia on DNA methylome and early pancreatic differentiation. Culture in hyperglycemic conditions disturbed the pancreatic lineage potential of hESC, leading to the downregulation of expression of pancreatic markers *PDX1*, *NKX6−1* and *NKX6−2* after in vitro differentiation. Genome-wide DNA methylome profiling revealed over 2000 differentially methylated CpG sites in hESC cultured in hyperglycemic condition when compared with those in control glucose condition. Gene ontology analysis also revealed that the hypermethylated genes were enriched in cell fate commitment. Among them, *NKX6−2* was validated and its hypermethylation status was maintained upon differentiation into pancreatic progenitor cells. We also established mouse ESC lines at both physiological glucose level (PG-mESC) and conventional hyperglycemia glucose level (HG-mESC). Concordantly, DNA methylome analysis revealed the enrichment of hypermethylated genes related to cell differentiation in HG-mESC, including *Nkx6−1*. Our results suggested that hyperglycemia dysregulated the epigenome at early fetal development, possibly leading to impaired pancreatic development.

## 1. Introduction

Diabetes is a global health concern. In a recent report, it was estimated that over 400 million patients were suffering from diabetes and the global prevalence of diabetes and impaired glucose tolerance would reach over 500 million in 20 years [1]. Type 2 diabetes (T2D) accounts for around 90% of the diabetic cases. Lifestyle and genetics are the two major risk factors leading to the disease. In addition, the *in-utero* environment, which plays a pivotal role in fetal development, is considered as another risk factor for T2D development in offspring. In fact, the concept of “Developmental origins of health and disease” has long been proposed, stating that alterations in the fetal environment had consequences in postnatal life, including later onset of non-communicable diseases [2,3]. Consistently, early studies demonstrated a strong association between fetal exposure to maternal diabetes and later occurrence of metabolic disorders such as obesity, glucose intolerance, and T2D in offspring [4,5,6].

T2D has been heavily linked with epigenetic dysregulation such as changes in the DNA methylome. For instance, several studies reported DNA methylome changes in pancreatic islets of T2D patients [7,8]. Upon onset of diabetes, *Pdx1*, an important transcription factor for pancreas development, is DNA hypermethylated in rats [9]. *Igf2* and *H19* are also hypermethylated in the pancreatic islets of adult offspring born from intrauterine hyperglycemic condition in mice [10]. More importantly, in vitro studies showed that pre-implantation embryos were vulnerable to epigenetic changes upon challenges by external stimuli. Hyperglycemic conditions reduce cell proliferation and blastocyst formation rates in animals [11,12]. In fact, there is a dynamic change in the DNA methylome during early embryogenesis from zygotic to post-implantation stages [13]. The early embryos are therefore susceptible to epigenetic changes induced by the intrauterine environment [14,15]. However, the exact mechanisms on how the changes in epigenetic regulation affect development of diseases remains largely unknown.

We have previously utilized human embryonic stem cell (hESC) as an in vitro model and demonstrated that hyperglycemic conditions dysregulated the histone methylation patterns, linking to impaired differentiation of definitive endoderm (DE), a progenitor of the pancreatic development process [16]. Here, we further showed that hyperglycemic conditions dysregulated the DNA methylome pattern in undifferentiated hESC. The hypermethylated genes were enriched in cell fate commitment, insulin secretion and glucose transport. More importantly, the dysregulated DNA methylome was concordant with gene expression patterns and impairment of early pancreatic differentiation. Our study provided insight on the impact of environmental insults on early developmental events, possibly leading to adult onset of diseases.

## 2. Results

### 2.1. Pancreatic Differentiation Potential from Human Embryonic Stem Cell (hESC) Was Disrupted in Hyperglycemic Culture Conditions

**Figure 1:**
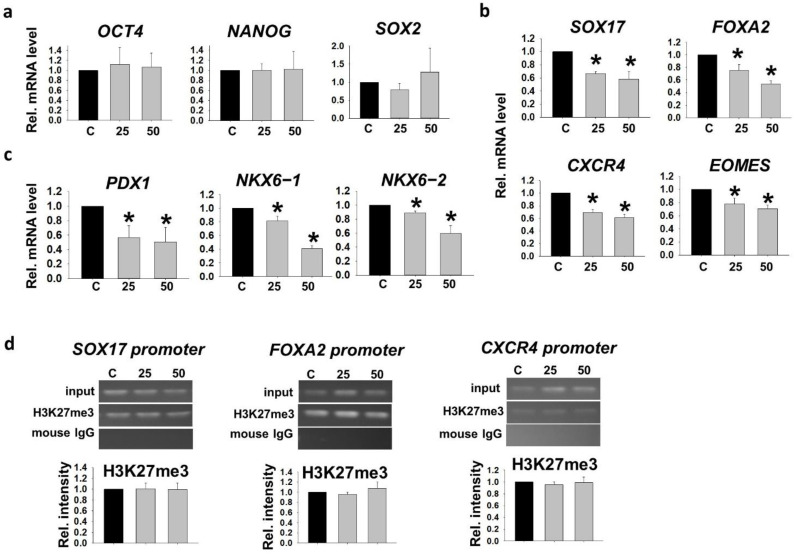
Human embryonic stem cell (hESC) cultured in hyperglycemic conditions showed disrupted pancreatic differentiation potential. (**a**) The relative mRNA levels of pluripotent markers (*OCT4*, *NANOG* and *SOX2*) in undifferentiated VAL3 under hyperglycemic conditions (C: 13.7 mM D-glucose) (*n* = 4). (**b**) The relative mRNA levels of definitive endoderm (DE) markers (*SOX17*, *FOXA2*, *CXCR4* and *EOMES*) in DE cells from different conditions of VAL3 (*n* = 4, * *p* < 0.05 compared to control, one-way ANOVA followed by Student–Newman–Keuls). (**c**) The relative mRNA levels of pancreatic progenitor (PP) markers (*PDX1*, *NKX6−1* and *NKX6−2*) in PP cells from different conditions of VAL3 (*n* = 4, * *p* < 0.05 compared to control, one-way ANOVA followed by Student–Newman–Keuls). (**d**) The H3K27me3 binding levels on the promoters of DE markers in undifferentiated VAL3 under hyperglycemic conditions (*n* = 3).

We showed previously that hyperglycemic conditions retained the repressive H3K27me3 marks on the promoters of pancreatic genes (*SOX17*, *FOXA2*, *CXCR4* and *EOMES*), leading to downregulation of their mRNA expression during DE differentiation from hESC [16]. By adopting our established hESC pancreatic progenitor cell differentiation model, we cultured a hESC line, VAL3 [17] in hyperglycemic conditions (25 mM and 50 mM D-glucose, 13.7 mM as control) for 5 passages. RT-qPCR analyses showed that the mRNA expression levels of pluripotent markers (*OCT4*, *NANOG* and *SOX2*) of the cells in these conditions were comparable (Figure 1a). The treated cells were then subjected to early pancreatic differentiation into DE and pancreatic progenitor (PP) cells under control glucose level. The 25 and 50 mM glucose-treated VAL3 had significantly lower expressions of DE markers (*SOX17*, *FOXA2*, *CXCR4* and *EOMES*) after 5 days of DE induction when compared to the control (Figure 1b). The effect was extended to the PP stage, with significantly lower expressions of PP markers (*PDX1*, *NKX6−1* and *NKX6−2*) in the hyperglycemia-treated group than the control (Figure 1c). We further examined the histone bivalency of the undifferentiated VAL3 cultured in the three conditions. Chromatin immunoprecipitation (ChIP)-PCR analyses revealed the presence of repressive H3K27me3 marks on the promoter of the DE markers, *SOX17*, *FOXA2* and *CXCR4* but the levels of the histone methylation were not affected (Figure 1d).

### 2.2. Hyperglycemia Altered Global DNA Methylome in hESC

**Figure 2:**
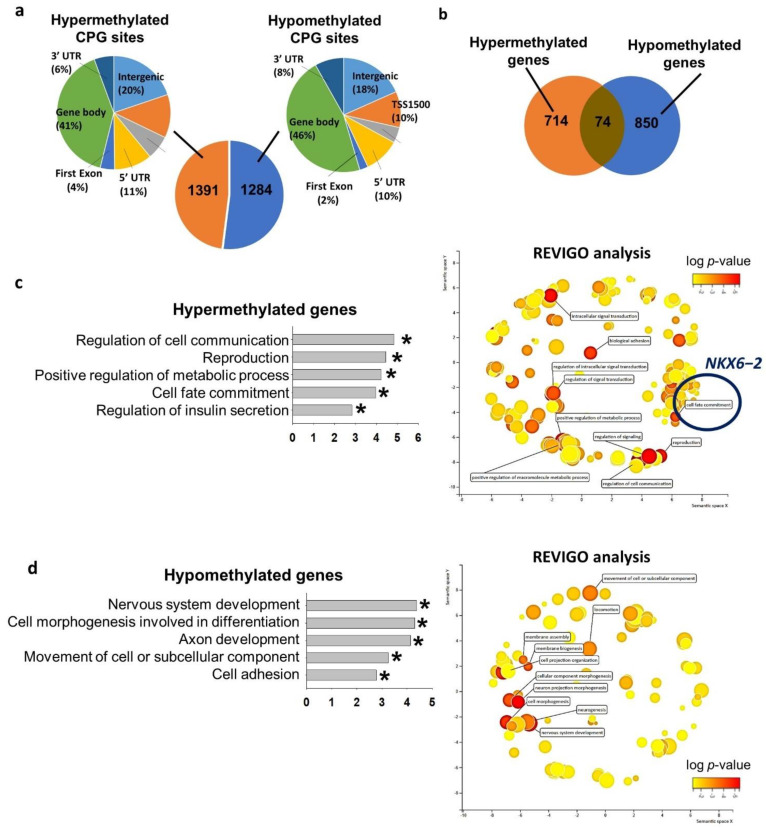
Hyperglycemia altered global DNA methylome in hESC. (**a**) Number of differentially methylated CpG sites in VAL3 under hyperglycemic condition (50 mM d-glucose; 13.7 mM basal level as control) and their genomic distribution. UTR: untranscribed region, TSS1500: 1500 bp upstream of transcription start site, TSS200: 200 bp upstream of transcription start site. (**b**) Number of differentially methylated genes in VAL3 under hyperglycemic condition. (**c**) (**left**) Gene ontology analysis of the hypermethylated genes. (* *p* < 0.05), (**right**) Cluster-plot of gene ontology terms processed with REVIGO analysis. Top 10 grouped gene ontology terms were shown. (**d**) (**left**) Gene ontology analysis of the hypomethylated genes. (* *p* < 0.05), (**right**) Cluster-plot of gene ontology terms processed with REVIGO analysis. Top 10 grouped gene ontology terms were shown.

We next studied the genome-wide DNA methylomes of VAL3 cultured in control glucose (13.7 mM) and hyperglycemic (50 mM) conditions using the Infinium HumanMethylation450 BeadChip array. The analysis revealed 1391 hypermethylated cytosines followed by guanine residues (CpG) sites and 1284 hypomethylated CpG sites in the hyperglycemia-treated VAL3 when compared to the control. The distribution of the CpG sites in different genomic regions were similar between hypermethylated and the hypomethylated regions (Figure 2a). The CpG sites were annotated to 714 hypermethylated and 850 hypomethylated genes induced by the hyperglycemic condition (Figure 2b). Gene ontology analyses revealed that the hypermethylated genes were related to cell communication, reproduction, regulation of metabolic process, cell fate commitment and regulation of insulin secretion (Figure 2c and Table S1). We subjected all significant gene ontology terms for REduce and Visualize Gene Ontology (REVIGO) analysis which grouped and clustered the biological terms based on nature of similarity [18]. The results confirmed that the most significant terms were intracellular signal transduction, reproduction, regulation of metabolic process, cell fate commitment (Figure 2c and Table 1). Among the genes clustered in the cell fate commitment term, *NKX6−2* was significantly downregulated during differentiation of the hyperglycemia-treated VAL3. On the other hand, the hypomethylated genes were enriched in nervous system development, cell morphogenesis involved in differentiation, axon development, cell movement and cell adhesion (Figure 2d and Table S1). REVIGO analysis revealed the enrichment of clustered Gene ontology (GO) terms such as neurogenesis, cellular component morphogenesis and movement of cell (Figure 2d and Table 1).

### 2.3. NKX6−2 Was Hypermethylated under Hyperglycemic Condition

**Figure 3:**
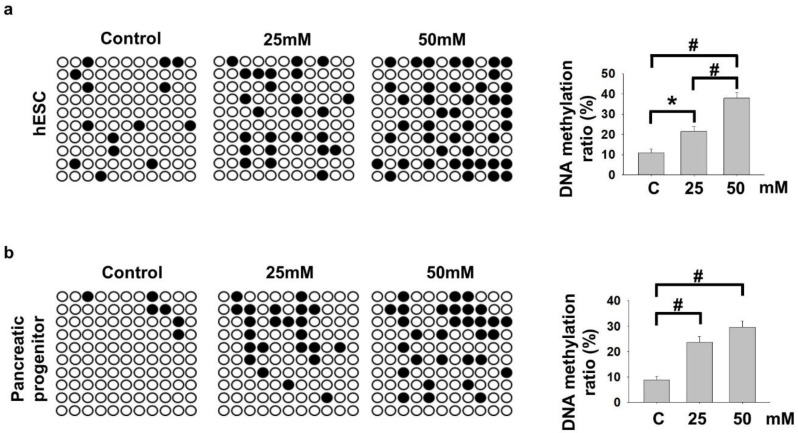
Hyperglycemia led to persistent hypermethylation of *NKX6−2* promoter. (**a**) Bisulfite sequencing results showing the DNA methylation status of *NKX6−2* promoter in undifferentiated VAL3 under different conditions. Black and white dots represented methylated and unmethylated CpG sites, respectively (*n* = 30, * *p* < 0.05, # *p* < 0.001, Chi-square test). (**b**) Bisulfite sequencing results showing the DNA methylation status of *NKX6−2* promoter in PP cells differentiated from VAL3 cultured in different conditions (*n* = 30, # *p* < 0.001, Chi-square test).

We and others [16,19] have demonstrated the importance of *NKX6−2* in regulating pancreatic progenitor differentiations. It was among the hypermethylated genes enriched in cell fate commitment and was down-regulated in PP cells differentiated from hyperglycemia-treated VAL3 (Figure 1c), suggesting potential epigenetic regulation of the gene during pancreatic differentiation. Using conventional bisulfite PCR, it was found that 25 mM and 50 mM glucose culture significantly induced DNA methylation levels of *NKX6−2* in the treated undifferentiated VAL3 (Figure 3a). More importantly, the hypermethylation status of *NKX6−2* in VAL3 with prior hyperglycemia (25 mM and 50 mM) treatment was maintained after induction of PP differentiation at a glucose level of 13.7 mM (Figure 3b).

### 2.4. Meta-Analysis of DNA Methylomes of Hyperglycemia Treated VAL3 with Dysregulated Methylomes of Human Pancreatic Islets from T2D Patients

**Figure 4:**
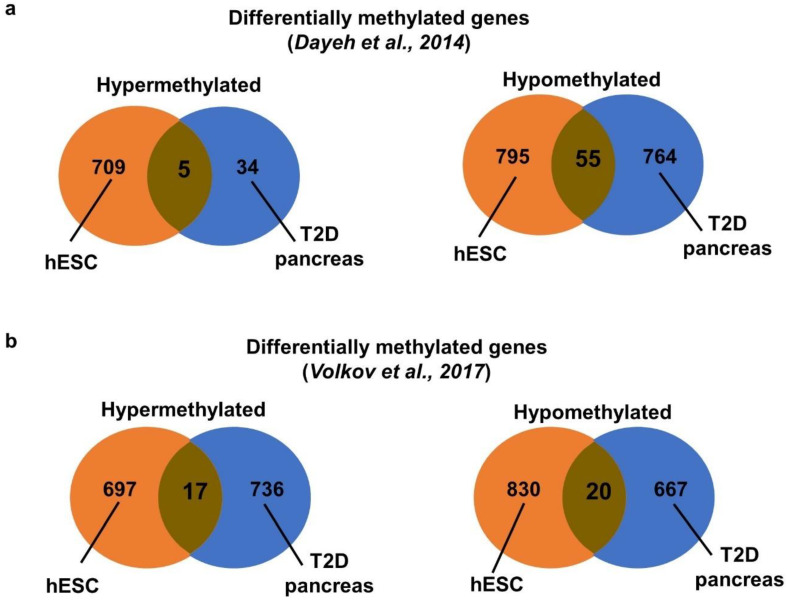
Meta-analysis with dysregulated methylome from pancreatic islets of T2D patients. Venn diagram showing the comparison of hypermethylated and hypomethylated genes between hyperglycemia-treated VAL3 and T2D pancreas from (**a**) Dayeh et al. [7] and (**b**) Volkov et al. [20].

We further compared our methylome data with two published methylome datasets of human pancreatic islets from T2D patients. One dataset was conducted with DNA methylation array [7] while the other with whole genome bisulfite sequencing [20]. The 2 studies shared only 1 hypermethylated (*CACNA1H*) and 23 hypomethylated genes (Table S2). We compared our methylome data with these datasets. The study of Dayeh and coworkers [7] identified mainly hypomethylated genes. We found five common hypermethylated and 55 hypomethylated genes in the hyperglycemia-treated VAL3 and the T2D pancreas (Figure 4a, Table S3). Interestingly, among the hypomethylated genes, *HDAC4* and *KCNQ1* were reported as T2D candidate genes. In contrast, the other dataset found similar numbers of differentially hypermethylated and hypomethylated genes in the T2D and the control pancreas [20]. We found 17 commonly hypermethylated and 20 hypomethylated genes in the hyperglycemia-treated VAL3 and the T2D pancreas (Figure 4b, Table S3). We next compared the gene ontology terms in the 3 datasets. Interestingly, similar hypermethylated terms, including secretion, regulation of transport and positive regulation of cell differentiation were identified in our hESC and Volkov’s datasets. On the other hand, similar hypomethylated terms, including cell adhesion, cell mobility and nervous system development, were identified in all 3 datasets (Tables S1 and S4). Only 1 hypermethylated (*CACNA1H*) and 1 hypomethylated (*S100A2*) gene were commonly identified in the 3 datasets (Table S3).

### 2.5. Conventional Hyperglycemic Culture Condition Dysregulated Mouse ESC DNA Methylome of Genes Related to Metabolism and Glucose Transport

The commonly used culture media for hESC and mESC contain supra-physiological glucose levels. For instance, the glucose levels in mTeSR1 (13.7 mM) [16], mouse embryonic fibroblast conditioned medium (25 mM) for hESC culture [21], and 2i/leukemia inhibitory factor (LIF) medium (25 mM) for mESC culture [22] are higher than the physiological level (around 5 mM) [23]. We therefore attempted to derive mESC line at physiological glucose level. The pluripotency of the physiological glucose (5.5 mM) derived mESC (PG-mESC) and the conventional hyperglycemic (25 mM) derived mESC (HG-mESC) were confirmed by spontaneous differentiation through embryoid body (EB) formation. RT-qPCR results showed that the differentiated PG-mESC and HG-mESC exhibited upregulation of ectoderm (*Sox1*) [24], mesoderm (*T*) [25] and endoderm (*Cdx2*) marker [26] expressions, and down-regulation of pluripotent markers (Oct4 and Nanog) expressions. No difference was found in the expression of *Sox1*, *T* and *Cdx2* in EBs between the PG-mESC and the HG-mESC (Figure S1a). The two types of mESC expressed immunoreactivity of mesoderm (muscle actin), endoderm (Cdx2) and ectoderm (beta-3 tubulin) markers in their EBs (Figure S1b) and had similar proportion of cardiac cells with rhythmic beating in the differentiating EBs (Figure S1c). These results demonstrated that the pluripotency of mESC derived at physiological glucose level (5.5 mM) was similar to those conventionally derived with supra-physiological glucose concentrations.

**Figure 5:**
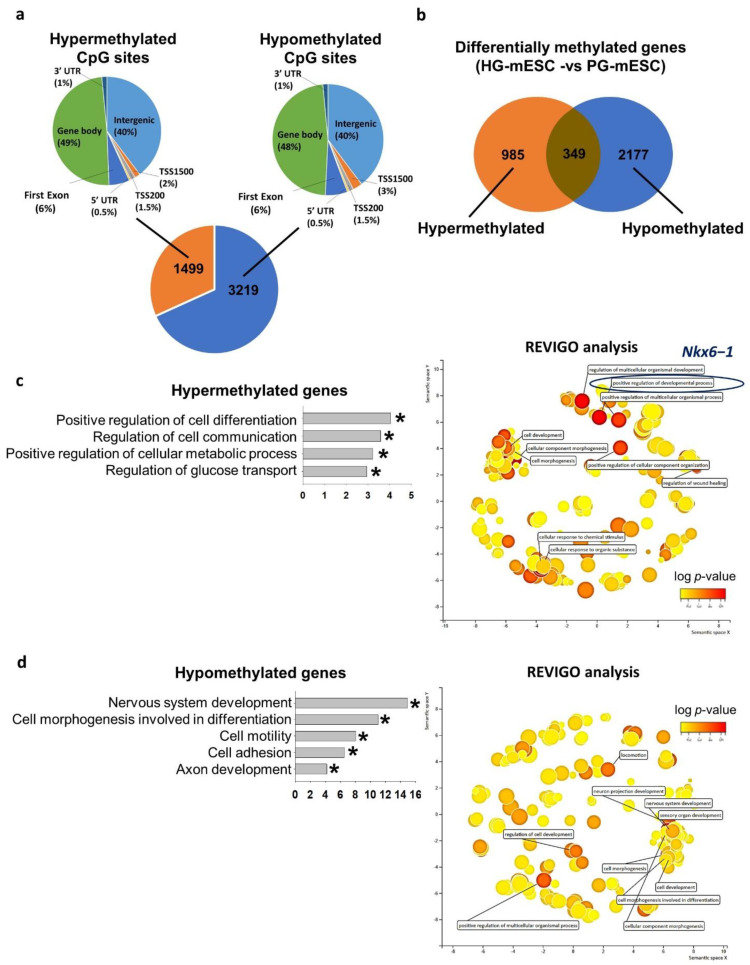
Conventional hyperglycemic culture condition dysregulated mouse ESC DNA methylome related to metabolism and glucose transport. (**a**) Number of differentially methylated CpG sites in mESC derived under hyperglycemic condition (HG-mESC: 25 mM), while the control was mESC derived under physiological glucose condition (PG-mESC: 5.5 mM) and their genomic distribution. UTR: untranscribed region, TSS1500: 1500 bp upstream of transcription start site, TSS200: 200 bp upstream of transcription start site. (**b**) Number of differentially methylated genes in HG-mESC. (**c**) (**left**) Gene ontology analysis of the hypermethylated genes. (* *p* < 0.05), (**right**) Cluster-plot of gene ontology terms processed with REVIGO analysis. Top 10 grouped gene ontology terms were shown. (**d**) (**left**) Gene ontology analysis of the hypomethylated genes. (* *p* < 0.05), (**right**) Cluster-plot of gene ontology terms processed with REVIGO analysis. Top 10 grouped gene ontology terms were shown.

We next compared the genome-wide DNA methylome status of PG-mESC and HG-mESC using reduced representation bisulfite sequencing (RRBS). When compared to PG-mESC, ~70% of the CpG sites were hypomethylated (3219) and ~30% of the sites were hypermethylated (1499) in the HG-mESC. Similar to hESC, the distribution of CpG sites in different genomic regions were similar between hypermethylated and hypomethylated CpG sties (Figure 5a). Concordantly, the number of hypomethylated genes (2177) were higher than that of hypermethylated (985) in the HG-mESC (Figure 5b). We subjected the differentially methylated genes to gene ontology analysis. The hypermethylated genes were enriched for cell differentiation, cell communication, regulation of metabolism and glucose transport (Table 2). REVIGO analysis indicated that the top biological terms clustered were regulation of developmental process and cellular response to organic substance. More importantly, *Nkx6−1*, the paralog of *Nkx6−2* was also found to be hypermethylated in the HG-mESC related to positive regulation of differentiation process (Figure 5c and Table 2). On the other hand, the hypomethylated genes were enriched in nervous system development, cell morphogenesis, cell motility, cell adhesion and axon development (Table 2). REVIGO analysis showed enrichment of terms including nervous system development, cell morphogenesis and locomotion (Figure 5d and Table 2).

We next compared the human and mouse methylome datasets. Interestingly, both datasets had high similarity in the gene ontology terms enriched. For instance, positive regulation of cell differentiation, metabolism and cell communication were commonly hypermethylated. Nervous system development, cell motility and cell adhesion were commonly hypomethylated (Table S1 and Table 2).

## 3. Discussion

In our previous study, we demonstrated that hyperglycemia during early pancreatic differentiation of hESC disrupted the histone methylation of key pancreatic markers, leading to the impairment of pancreatic progenitor differentiation [16]. In this study, hESC with prior exposure to hyperglycemic conditions also exhibited disrupted pancreatic differentiation even when differentiation was induced at a conventional glucose level. The results revealed a critical concept that transient high glucose exposure in embryos was sufficient for epigenetic dysregulation, thus pre-disposing to defects in subsequent pancreatic differentiation and development.

Hyperglycemic treatment of mouse zygotes at glucose level (28 mM) found in diabetes significantly reduced the number of live pups [27]. However, the windows of exposure causing the detrimental effects to development remains to be elucidated. Early pre-implantation embryos are vulnerable to external stimuli and are genetically [28] and epigenetically [29] unstable. In vitro fertilization was reported to increase the change of genomic imprinting defects such as Beckwith–Weidemann syndrome (BWS) through altered DNA methylation [29]. We previously demonstrated that hyperglycemia altered histone bivalency in the differentiating cells during DE formation [16]. The present data suggested that such a change in histone bivalency was absent in hyperglycemic-treated undifferentiated hESC. However, DNA methylome was dysregulated in hESC under hyperglycemic culture conditions and linked to the impaired pancreatic differentiation. The discrepancy could be due to the fact that most of the lineage-specific genes were already bivalently marked at an undifferentiated state. The repressive H3K27me3 marks were only removed from the pancreatic gene promoters during the differentiation process and, therefore, affected by hyperglycemia treatment in our previous study [30]. On the other hand, DNA methylation status varied from unmethylated to fully methylated states in hESC [31]. Whether hypeglycemia interrupted the interplay between DNA methylation and histone bivalency during early developmental process requires further study.

Gene ontology analysis identified hypermethylation of genes related to cell fate commitment and regulation of insulin secretion in the hyperglycemia-treated VAL3. Coincidently, the expression of *NKX6−2* was also significantly reduced upon differentiation in such condition. *Nkx6−2* can partially compensate for *Nkx6−1* function in the formation of mouse pancreatic α cells [32] important for glucose homeostasis. Upon stimuli such as tissue injury or diseased state, the α cells can be transdifferentiated as a new source of pancreatic β cells [33,34]. It was thus postulated that the silencing of *NKX6−2* might result in pancreas malfunction [32]. The persistent maintenance of *NKX6−2* hypermethylation status from undifferentiated VAL3 to differentiated pancreatic progenitor cells suggests that early dysregulated DNA methylation may be maintained during development, possibly linking to future T2D development.

Comparisons of DNA methylome between normal and T2D islets [7,20] provide valuable information on the pathogenesis of the disease. In an attempt to identify epigenetic regulators linking to the etiology of T2D, we cross-compared our DNA methylome data with the published datasets. Interestingly, the DNA methylome of T2D pancreatic islets were mainly hypomethylated which contributed to 96–97% of the differentially methylated CpG sites [7,8]. However, the proportion of hypermethylated and hypomethylated genes were similar in another study [20], which was in line with our methylome data utilizing hESC. One reason for the discrepancy might be attributed to the fact that T2D islets were already in a diseased state with extensive epigenetic dysregulation, while hESC were in an early and non-differentiated state. Another reason could be the differences in sequencing depth, as whole genome bisulfite sequencing (WGBS) assayed 50 times more CpG sites than DNA methylation arrays. It would be worthwhile to opt for WGBS in the future to identify more potential candidate genes affected by hyperglycemia during development.

We identified only 1 hypermethylated (*CACNA1H*) and 1 hypomethylated (*S100A2*) gene shared in the three studied datasets. *CACNA1H* encodes the voltage-dependent T-type calcium channel alpha-1H subunit. The mutation of *CACNA1H* increases aldosterone production and is heavily associated with primary aldosteronism [35,36]. Since primary aldosteronism is one of the reported complications of T2D patients with resistant hypertension [37], we speculate the changes in methylation of *CACNA1H* in pancreatic cells is the outcome of T2D. Overexpression of *S100A2* promotes glycolysis and proliferation of colorectal cancer cells [38]. Its function is mainly reported in epithelial cells such as keratinocytes [39] and melanoma cells [40], but its role in pancreatic differentiation is not known. In comparison with individual datasets, 2 hypomethylated genes (*HDAC4* and *KCNQ1*) were commonly found in the hyperglycemia-treated VAL3 and the Dayeh’s study. Both genes have been associated with T2D. *HDAC4* mutation induces pancreatic β-cell loss and decrease in insulin secretion, leading to diabetic phenotype in mice [41]. *KCNQ* mutation is positively correlated with T2D susceptibility [42] via an unknown mechanism. Since DNA hypomethylation is usually correlated with an increase in gene expression [43], the hypomethylated states of the two genes in both datasets might suggest their roles in disease etiology. The low number of common genes identified in our methylomes and the patients’ islets suggested that hyperglycemia-induced hypermethylation in undifferentiated hESC might not be maintained in terminally differentiated T2D islets. The roles of hyperglycemia-induced methylation changes during the differentiation of pancreatic progenitor and their association with the onset on T2D later in life warrant future study.

The conventional human and mouse ESC culture conditions involved the utilization of culture medium in supra-physiological glucose levels. Several studies attempted to compare the effects of physiological and hyperglyecmia conditions on ESC maintenance and differentiation potency. For instance, one study reported hyperglycemic culture conditions (25 mM and 55 mM) induced reactive oxygen species (ROS), leading to reduced proliferation of mESC when compared to physiological conditions (1.1 and 5.5 mM) [44]. Another study also demonstrated mESC had reduced cardiac differentiation potency in hyperglyecmic condition (25 mM) relative to those in physiological level (5 mM) [45]. However, since the ESC used in these studies were derived and cultured in conventional high glucose condition (25 mM) for long periods of time, the epigenome including the DNA methylome might have already been modified when the ESC was adapted to culture in such conditions. In view of that, an earlier study derived mESC at physiological and conventional high glucose conditions. It was found that mESC derived in physiological glucose levels expressed glucose transporter *Glut2* similar to that in pre- and early post-implantation mouse embryos, and the expression was higher than those derived in high glucose [46]. Since *Glut2* is one of the glucose transporters expressed during early embryo stage, they suggested that mESC derived in physiological glucose levels had similar metabolic mechanism as pre-implantation embryos. However, they did not characterize both mESC extensively.

In this study, we also derived PG-mESC and HG-mESC from mouse blastocysts at physiological (5.5 mM) and conventional (25 mM) glucose levels, respectively. This is the first study to extensively characterize these mESC. To our surprise, the two mESC lines showed distinct DNA methylation patterns; HG-mESC had 1499 differentially hypermethylated and 3219 hypomethylated CpG sites when compared to PG-mESC. Importantly, gene ontology analyses revealed that genes related to cellular differentiation and glucose transport were hypermethylated in the HG-mESC. Among the enriched terms, cellular differentiation was both hypermethylated in our human and mouse datasets. In fact, an important pancreatic transcription factor *Nkx6−1* was found to be hypermethylated in the HG-mESC. As mentioned above, *Nkx6−1* works together with its paralog *Nkx6−2* during pancreas organogenesis. *Nkx6−1* depleted mouse embryos exhibited a remarkable reduction in the number of insulin-expressing cells [47]. The hypermethylation of *Nkx6−1* in HG-mESC further supported the epigenetic impairment imposed by hyperglycemia in the human model. We found only a small proportion of overlapping genes (5%) between our hyperglycemia-treated VAL3 and HG-mESC data, possibly because the metabolic pathways and expressions of glucose transporters were different between hESC and mESC.

Due to ethical issues, the study on the effects of maternal hyperglycemia on the epigenetic alteration in peri-implantation embryos is very difficult. Although hESC is a valuable experimental model for studying early pancreatic development in the current study, it suffered from some limitations. First, hESCs were derived and maintained in glucose levels higher than that of physiological levels. Human ESC derivation from human embryos in a normal physiological range of glucose might be a future approach to confirm the current findings in humans. Second, the pancreatic progenitors differentiated from hESC only represent very early progenitor cells. Whether the detrimental effects of early hyperglycemia exposure affect the pancreatic function, including the production of insulin producing cells, is lacking in the current study model. From this study, several pathways were identified in both hESC and mESC that were hypermethylated in hyperglyecmia conditions. Those pathways included cell fate commitment, cell communication and glucose transport. Further studies are required to uncover the candidate genes involved in those pathways and investigate the causal relationship between hypermethylation and T2D development. Several studies have reported the gene-specific DNA methylation or de-methylation through CRISPR/Cas9 approach [48,49]. The functional roles of the candidate genes on pancreatic differentiation can also be investigated by gene knockout approach in hESC or mESC. The roles of maternal hyperglycemia conditions leading to later development of T2D can also be explored in vivo by establishing chimeric mouse models using PG-mESC and HG-mESC, where differentiated mESC can be traced and isolated from the pancreatic islets for further molecular and functional analysis.

In conclusion, this study demonstrated that hyperglycemia extensively dysregulated the DNA methylomes of hESC and mESC. The hypermethylated genes were enriched for the processes of pancreas differentiation, insulin secretion and glucose transport. More importantly, we demonstrated that *NKX6−2*, an important pancreatic progenitor marker, to be persistently hypermethylated even after differentiation. Our methylome pattern at the early undifferentiated stage may provide insights into how the dysregulated epigenome at early development may lead to the outcome of T2D development in the future.

## 4. Materials and Methods

### 4.1. Human Embryonic Stem Cells (ESC) Culture and Hyperglycemic Conditions

Human ESC line, VAL3 [17] was obtained from the Centro de Investigación Príncipe Felipe (CIPF) in Valencia, Spain. VAL3 was cultured in mTeSR1 medium (Stemcell Technologies, Vancouver, VC, Canada) as described [16]. The glucose level of mTeSR1 medium was 13.7 mM. For hyperglycemic conditions, D-glucose (Sigma-Aldrich, Burlington, MA, USA) was supplemented into the mTeSR1 medium to levels of 25 mM and 50 mM.

### 4.2. Differentiation of hESC into Definitive Endoderm and Pancreatic Progenitor Cells

VAL3 was differentiated into DE cells using the STEMdiff Definitive Endoderm Kit (Stemcell Technologies) according to manufacturer’s instructions with slight modifications. Briefly, VAL3 was seeded at 2.1 × 105 cells/cm^2^ on Matrigel-coated plate in mTeSR1 medium supplemented with 10 µM Y-27632 (Stemcell Technologies). The medium was replaced with the differentiation medium on the next day. Medium was changed daily for 5 days. VAL3 was then further differentiated into pancreatic progenitor cells according to our published protocol [16]. 

### 4.3. Bisulfite Sequencing

Bisulfite sequencing was conducted as described [50]. Briefly, extracted DNA samples were bisulfite converted using the Epitect Bisulfite Kit Protocol (Qiagen, Düsseldorf, Germany). The sequencing service was provided by Tech Dragon Ltd., Hong Kong. The PCR primers were listed as follows: *NKX6−2* forward: GGTTTGGAAAAGTTATTTG; *NKX6−2* reverse: AAACTACACCTCAATAAAACC.

### 4.4. Derivation of Mouse Embryonic Stem Cell (mESC) Lines

Mouse ESC lines were derived from blastocysts of ICR mice. Female ICR mice at 6–8 weeks old were super-ovulated by injection with 5 IU of pregnant mare serum gonadotropin (PMSG) and 5 IU of human chorionic gonadotropin (hCG; Sigma-Aldrich) at 48 h interval. Pregnant mice were selected after successful mating. The blastocysts were flushed from the uterus on 3.5 dpc using M2 medium (Sigma-Aldrich). The zona pellucida of the blastocysts were removed with acid tyrode solution (Sigma-Aldrich). The zona-free blastocysts were then placed in mouse embryonic fibroblast (MEF) feeder cells previously inactivated by mitomycin-c treatment. The culture medium was 2i/LIF medium consisted of DMEM low glucose (5.5 mM, Thermo Fisher Scientific, Waltham, MA, USA) supplemented with 3 µM CHIR99021 (R&D Systems, Minneapolis, MN, USA), 1 µM PD0325901 (R&D Systems), 1000 units/mL leukemia inhibitory factor (LIF), 15% Knockout serum replacement, 1 × penicillin and streptomycin, 1 × L-glutamine, 1 × non-essential amino acids, 1 × B27 supplement, 1 × N2 supplement (all from Thermo Fisher Scientific), 1 mM sodium pyruvate (Sigma-Aldrich) and 0.1 mM β-mercaptoethanol (Sigma-Aldrich). The culture was maintained until outgrowths were observed 6–8 days after seeding. The animal experiments were performed in accordance with the Committee on the Use of Live Animals in Teaching and Research, The University of Hong Kong (CULATR, HKU). The established cell line was cultured in 2i/LIF medium as described [16]. For hyperglycemic conditions, DMEM high glucose (25 mM, Thermo Fisher Scientific) in 2i/LIF medium was used for the cell culture.

### 4.5. Embryoid Body (EB) Formation from mESC

mESC was differentiated into embryoid bodies (EBs) using our modified hanging drop method [51]. PG- and HG-mESC were seeded at a density of 500 cells/20 µL drop on the lid of the Petri dish. After 2 days aggregation in the inverted lid, the EBs were transferred to 96-well ultra-low attachment plates (Corning, New York, NY, USA) in EB medium (DMEM high glucose supplemented with 15% FBS, 1 × penicillin and streptomycin, 1 × non-essential amino acids and 0.1 mM β-mercaptoethanol). After 3 days, the EBs were transferred to 48-well plates pre-coated with 0.1% gelatin for attachment and continued culture.

### 4.6. Genome-Wide Methylation Analysis

Genome-wide methylation analysis was conducted on VAL3 using the Infinium HumanMethylation450 BeadChip array for detecting human methylome (Illumina, San, Diego, CA, USA). Briefly, 750 ng of genomic DNA was hybridized to the BeadChips, followed by single base extension and staining of BeadChips. The data were then analyzed by GenomeStudio (Illumina). A differential score of >13 or <−13 was considered as significantly hypermethylated or hypomethylated, respectively. The genome-wide methylation analysis was conducted on mESC using reduced representation bisulfite sequencing (RRBS). Briefly, 2.5 µg of genomic DNA was digested overnight with restriction enzyme MspI (New England Biolabs, Ipswich, MA, USA). The digested DNA samples were used to construct libraries, followed by bisulfite conversion with the EZ DNA Methylation-Lightning Kit (Zymo Research, Irvine, CA, USA). The Illumina HiSeq SBS Kit v4 (Illumina) was used for paired-end 101 bp sequencing. Sequence reads were trimmed by the Trim Galore, followed by alignment to the mouse genome mm10 by Bismark, and annotation of methylation sites by HOMER. The differentially methylation analysis was conducted using methylKit [52]. A methylation difference of >25% with *p*-value of <0.05 was considered statistically significant. The sequencing and data analysis services were provided by the Centre for PanorOmic Sciences, the University of Hong Kong. The downstream gene ontology analysis for both hyperglycemia-treated VAL3 and HG-mESC were performed by the Database for Annotation, Visualization and Integrated Discovery (DAVID) platform (version 6.8, National Institutes of Health (NIH), Bethesda, MD, USA). In addition, REduce and Visualize Gene Ontology (REVIGO, Rudjer Boskovic Institute, Zagreb, Croatia) was used for condensation and clustering of gene ontology terms [18].

### 4.7. Quantitative Polymerase Chain Reaction (qPCR)

Total RNAs were extracted by the mirVana Protein and RNA Isolation System (PARIS) Kit (Thermo Fisher Scientific) and cDNA conversion was performed by the TaqMan Reverse Transcription kit (Thermo Fisher Scientific). Real time qPCR using the TaqMan Gene Expression Assay was performed in an Applied Biosystems 7500 Real-Time PCR System (Thermo Fisher Scientific). Quantification of gene expression levels were determined by the 2^−ΔΔCt^ method. The following probes were used: *OCT4* (assay ID: Hs04260367_gH, Mm03053917_g1), *NANOG* (assay ID: Hs02387400_g1, Mm02019550_s1), *SOX2* (Hs01053049_s1), *SOX17* (Hs00751752_s1, Mm00488363_m1), *FOXA2* (Hs05036278_s1), *CXCR4* (Hs00607978_s1), *EOMES* (Hs00172872_m1), *PDX1* (Hs00236830_m1), *NKX6−2* (Hs00752986_s1), *Sox1* (Mm00486299_s1), *T* (Mm00436877_m1).

### 4.8. Chromatin Immunoprecipitation (ChIP)

Chromatin immunoprecipitation (ChIP) was performed as previously described [16]. The primers used were as follows: SOX17 forward: GAATGGACGCTCGGTATGTT, *SOX17* reverse: GAGACTCGAAAAGCCGTCTG, *FOXA2* forward: GAGCCTCCACATCCAAACAC, *FOXA2* reverse: CAGCAGCTCTTGGGTTCAA, *CXCR4* forward: TCCACTTTAGCAAGGATGGAC, *CXCR4* reverse: TCCCAGAGGCATTTCCTAAG. The antibodies used were mouse anti-H3K27me3 (ab6002, Abcam, Cambridge, UK), rabbit anti-H3K4me3 (ab8580, Abcam), normal mouse IgG (sc2025, Santa Cruz) and normal rabbit IgG (12370, Millipore, Burlington, MA, USA).

### 4.9. Immunofluorescent Staining

Cells were fixed in 4% PFA and permeabilized with 0.1% Triton X-100 (Sigma-Aldrich). The antibodies against muscle actin (M0635, Agilent, Santa Clara, CA, USA), beta-3 tubulin (MA1-118, Thermo Fisher Scientific), CDX2 (12306, Cell Signaling Technology, Danvers, MA, USA), goat anti-mouse IgG, Alexa Fluor 568 (H + L) (A-11019, Thermo Fisher Scientific), goat anti-rabbit IgG, Alexa Fluor 568 (H + L) (A-11036, Thermo Fisher Scientific) were used. The nuclei were stained with Hoechst 33,258 (Thermo Fisher Scientific). Images of the stained cells were captured by using a confocal microscope (LSM 700, Carl Zeiss AG, Oberkochen, Germany) at the Centre for PanorOmic Sciences, the University of Hong Kong.

### 4.10. Statistical Analysis

Statistical analysis was conducted using the SigmaPlot 12.0 software (Systat Software, San Jose, CA, USA). The statistical tests used included *t*-test, one-way ANOVA followed by Student–Newman–Keuls, Mann–Whitney Rank Sum test or Chi-square test where appropriate. The data in the plots were represented as the mean ± standard error of mean (SEM). A *p*-value < 0.05 was considered statistically significant.

## Figures and Tables

**Table 1:** Gene ontology and REVIGO terms of differentially methylated genes in hESC cultured in hyperglycemic (50 mM) condition compared to basal glucose (13.7 mM) condition.

**Hypermethylated Genes**			
	**Term**	**Gene** **Count**	***p*-Value**
GO terms	GO:0010646-egulation of cell communication	136	1.49 × 10^−5^
	GO:0000003-reproduction	72	3.60442 × 10^−5^
	GO:0009893-positive regulation of metabolic process	134	6.12 × 10^−5^
	GO:0045165-cell fate commitment	21	1.10 × 10^−4^
	GO:0050796-regulation of insulin secretion	15	0.001501577
REVIGO	GO:0023051-regulation of signaling	139	0.006234194
	GO:0009966-regulation of signal transduction	126	0.006947825
	GO:0035556-intracellular signal transduction	123	0.007549951
	GO:0010646-regulation of cell communication	136	0.008015438
	GO:0000003-reproduction	72	0.011758664
	GO:0010604-positive regulation of macromolecule metabolic process	128	0.012049471
	GO:0009893-positive regulation of metabolic process	134	0.014799489
	GO:0022610-biological adhesion	84	0.014802392
	GO:0045165-cell fate commitment	21	0.019097456
	GO:1902531-regulation of intracellular signal transduction	83	0.019839541
**Hypomethylated genes**			
	**Term**	**Gene** **Count**	***p*-Value**
GO terms	GO:0007399-nervous system development	121	4.14929 × 10^−5^
	GO:0000904-cell morphogenesis involved in differentiation	53	4.89 × 10^−5^
	GO:0061564-axon development	36	7.21 × 10^−5^
	GO:0006928-movement of cell or subcellular component	98	5.51 × 10^−4^
	GO:0007155-cell adhesion	91	0.001670345
REVIGO	GO:0048812-neuron projection morphogenesis	139	0.004559117
	GO:0000902-cell morphogenesis	126	0.01148067
	GO:0007399-nervous system development	123	0.012500011
	GO:0030030-cell projection organization	136	0.014020097
	GO:0032989-cellular component morphogenesis	72	0.017652818
	GO:0044091-membrane biogenesis	128	0.022253644
	GO:0022008-neurogenesis	134	0.032864231
	GO:0071709-membrane assembly	84	0.033846
	GO:0006928-movement of cell or subcellular component	21	0.038433186
	GO:0040011-locomotion	83	0.046574919

**Table 2:** Gene ontology and REVIGO terms of differentially methylated genes in HG-mESC compared to PG-mESC.

**Hypermethylated Genes**			
	**Term**	**Gene** **Count**	***p*-Value**
GO terms	GO:0045597-positive regulation of cell differentiation	62	9.01 × 10^−5^
	GO:0010646-regulation of cell communication	140	2.56 × 10^−4^
	GO:0031325-positive regulation of cellular metabolic process	137	6.07 × 10^−4^
	GO:0010827-regulation of glucose transport	11	0.00112593
REVIGO	GO:0051094-positive regulation of developmental process	82	0.002840517
	GO:2000026-regulation of multicellular organismal development	112	0.002906425
	GO:0061041-regulation of wound healing	18	0.006799059
	GO:0071310-cellular response to organic substance	118	0.006814995
	GO:0048468-cell development	125	0.007998872
	GO:0051240-positive regulation of multicellular organismal process	94	0.008416542
	GO:0032989-cellular component morphogenesis	84	0.008509034
	GO:0070887-cellular response to chemical stimulus	139	0.008815601
	GO:0051130-positive regulation of cellular component organization	77	0.01068532
	GO:0000902-cell morphogenesis	78	0.011784721
**Hypomethylated Genes**			
	**Term**	**Gene** **Count**	***p*-Value**
GO terms	GO:0007399-nervous system development	291	1.22096 × 10^−15^
	GO:0000904-cell morphogenesis involved in differentiation	124	9.93 × 10^−12^
	GO:0048870-cell motility	171	8.82 × 10^−9^
	GO:0007155-cell adhesion	187	3.11 × 10^−7^
	GO:0061564-axon development	63	6.29 × 10^−5^
REVIGO	GO:0007399-nervous system development	291	3.33609 × 10^−7^
	GO:0048468-cell development	286	1.76681 × 10^−6^
	GO:0000902-cell morphogenesis	177	1.27546 × 10^−5^
	GO:0032989-cellular component morphogenesis	187	1.4186 × 10^−5^
	GO:0000904-cell morphogenesis involved in differentiation	124	1.66519 × 10^−5^
	GO:0051240-positive regulation of multicellular organismal process	204	4.87473 × 10^−5^
	GO:0031175-neuron projection development	131	0.000104272
	GO:0007423-sensory organ development	95	0.000163232
	GO:0040011-locomotion	192	0.00016505
	GO:0060284-regulation of cell development	138	0.00018271

## Data Availability

All data are contained within this manuscript.

## Supplementary Materials

The following are available online at https://www.mdpi.com/article/10.3390/ijms221910729/s1.
