# Supplementary material for: Hyperglycemia Altered DNA Methylation Status and Impaired Pancreatic Differentiation from Embryonic Stem Cells
Source: Int J Mol Sci. 2021 Oct 3;22(19):10729. doi: 10.3390/ijms221910729 (PMC8509790; doi:10.3390/ijms221910729)
Supplement: Supplementary file 1 [file ijms-22-10729-s001.zip › Figure S1, Table S2, Table S3.pdf]

International Journal of Molecular Sciences

MDPI

Supplementary materials

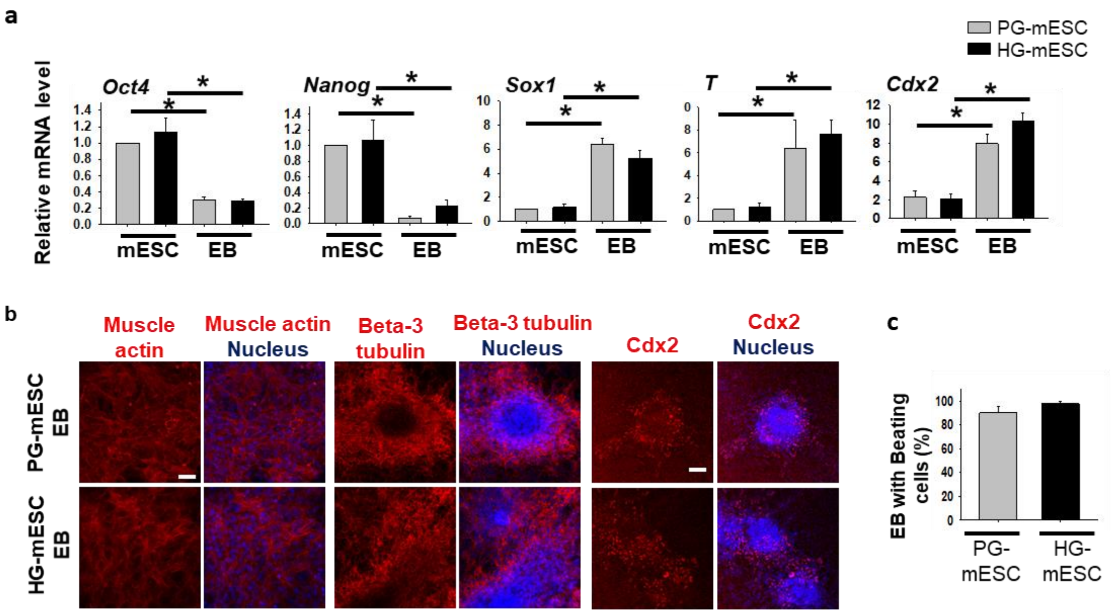

Figure S1. mESC derived from physiological glucose level was pluripotent. (**a**) The relative mRNA levels of pluripotent markers (*Oct4* and *Nanog*) and differentiation markers (*Sox1*, *T* and *Cdx2*) in undifferentiated state and spontaneously differentiated embryoid body (EB) from mESC derived under physiological glucose level (PG-mESC: 5.5mM) and hyperglycemia level (HG-mESC: 25mM) respectively ($n = 3$, $*p < 0.05$, t-test). (**b**) Immunofluorescent staining of muscle actin, beta-3 tubulin and Cdx2 in EB differentiated from PG- and HG-mESC. Scale bar: 100μm. (**c**) The rhythmic beating rate of EB differentiated from PG- and HG-mESC.

Table S2. List of common differentially methylated genes from Dayeh et al., 2014 [7] and Volkov et al., 2017 [20].

	**Dayeh et al., 2014** [7] **and Volkov et al., 2017 [20]**
**Commonly hypermethylated**	CACNA1H
**Commonly hypomethylated**	BCOR, PTPRN2, FAM135B, FEV, NPHP4, SRGAP3, C2orf72, MYOF, LRRC8D, FAM155A, SLC39A11, RORA, S100A2, S100A14, SBF2, BANP, GNA12, FAM20C, CRYBA2, TNFRSF11B, EML5, SH3RF1, CACNA1C

Table S3. List of common differentially methylated genes from hyperglycemia-treated VAL3, Dayeh et al., 2014 [7] and Volkov et al., 2017 [20].

	**Hyperglyecmia-treated VAL3 and Dayeh et al., 2014** [7]	**Hyperglyecmia-treated VAL3 and Volkov et al., 2017 [20]**
**Commonly hypermethylated**	NCOR2, ZFHX3, ITGB4, KCNQ4, ***CACNA1H***	MIR373, BAG3, ZBTB46, CATSPERG, SPEG, MTMR1, MIR372, IFITM2, INPP5A, SIDT1, DSCAML1, BCYRN1, ***CACNA1H,*** RPS6KA2, HIST1H2AB, ARX, CNKSR3
**Commonly hypomethylated**	EIF4E, NCKAP5, RNF39, CREB5, NHS, GXYLT1, PBX1, CAMTA1, GPR158, CUX2, TBC1D8, AFF1, PALLD, PLXNA1, ITGB5, TEAD3,	LMF1, KCNIP1, FAM19A5, ZNF333, COL21A1, NXN, COBL, DPP4, RMST, SLC22A16, LRP2BP, PLIN3, KIF2A, GPR63, PCDHA2,

	HDAC4, FAM190B, SMG6, LRP5, FOXP1, CORO1C, TBC1D14, MGMT, PDE7B, WWOX, PTK2B, GMDS, MAN1C1, ARHGAP26, FNDC3B, ARHGAP10, JAK3, NSMCE1, TANK, EFEMP1, IFI44L, EPS8, TNFAIP8, SLC4A4, SEMA5A, HIPK2, SGK3, ATP11A, MCC, LPP, DIRC3, SOX5, ***S100A2***, PSD3, KCNQ1, SPAG16, C18orf1, OSBPL6, TRPM1	PTPRG, ***S100A2***, MIPOL1, GALNTL6, PCDHA1
